# Association between LEPR, FTO, MC4R, and PPARG-2 polymorphisms with obesity traits and metabolic phenotypes in school-aged children

**DOI:** 10.1007/s12020-018-1587-3

**Published:** 2018-04-20

**Authors:** Sílvia M. Almeida, José M. Furtado, Paulo Mascarenhas, Maria E. Ferraz, José C. Ferreira, Mariana P. Monteiro, Manuel Vilanova, Fernando P. Ferraz

**Affiliations:** 1 Centro de Genética Médica e Nutrição Pediátrica Egas Moniz, Campus Universitário, Monte da Caparica, Portugal; 2 Instituto Universitário Egas Moniz, Campus Universitário, Monte da Caparica, Portugal; 3 Clinical and Experimental Endocrinology Group, Unit for Multidisciplinary Research in Biomedicine UMIB, ICBAS, University of Porto, Porto, Portugal; 4 Instituto de Investigação e Inovação em Saúde, and IBMC–Instituto de Biologia Molecular e Celular, Universidade do Porto, Porto, Portugal; 5 Instituto de Ciências Biomédicas de Abel Salazar, Universidade do Porto, Porto, Portugal

**Keywords:** Polymorphisms, Anthropometry, Body fat, Biochemical parameters, Children

## Abstract

**Purpose:**

Evaluate the relationship of leptin receptor (LEPR) rs1137101, fat mass obesity-associated (FTO) receptors 9939609, melanocortin-4 receptors (MC4R) rs2229616 and rs17782313, and proliferator-activated receptor-gamma (PPARG) rs1801282 with clinical and metabolic phenotypes in prepubertal children.

**Research question:**

What is the effect of polymorphisms on clinical and metabolic phenotypes in prepubertal children?

**Methods:**

A cross-sectional descriptive study was performed to evaluate anthropometric features, percentage body fat (%BF), biochemical parameters, and genotype in 773 prepubertal children.

**Results:**

FTO rs9939609 was associated with an increase in body mass index (BMI) and BMI *z*-score (zBMI). MC4R rs17782313 was associated with a decrease in BMI and +0.06 units in zBMI. LEPR, and PPARG-2 polymorphisms were associated with decreases in BMI and an increase and decrease units in zBMI, respectively. The homozygous SNPs demonstrated increases (FTO rs993609 and MC4R rs17782313) and decreases (LEPR rs1137101, PPARG rs1801282) in zBMI than the homozygous form of the major allele. In the overweight/obese group, the MC4R rs17782313 CC genotype showed higher average weight, zBMI, waist circumference, waist-circumference-to-height ratio, and waist-hip ratio, and lower BMI, mid-upper arm circumference, calf circumference, and %BF (*P*< 0.05). FTO rs9939609 AT and AA genotypes were associated with lower triglycerides (*P* < 0.05).

**Conclusions:**

We showed that MC4R rs17782313 and FTO rs9939609 were positively associated with zBMI, with weak and very weak effects, respectively, suggesting a very scarce contribution to childhood obesity. LEPR rs1137101 and PPARG-2 rs1801282 had weak and medium negative effects on zBMI, respectively, and may slightly protect against childhood obesity.

## Introduction

Obesity prevalence has increased during the past century and the World Health Organization (WHO) estimates that the number of overweight/obese young children will reach 70 million in 2025 [1]. Obesity is a chronic disease with multifaceted etiology [2, 3]. Socioeconomic changes during the last decades have contributed to these phenomena, including the increased availability of high-fat foods and generalized adoption of sedentary lifestyles. Furthermore, there is evidence that genes play an important role in the rise of obesity [2, 3]. Heritability is estimated to account for 40–90% of the population adiposity variation. Seemingly, the presence of single nucleotide polymorphisms (SNPs) offer a protective factor in the development of non-communicable diseases, such as obesity related diseases [4–6]. With the development of high-throughput genotyping techniques, new approaches such as genome-wide linkage and genome-wide association studies (GWAS) have been used to understand genetic influences in obesity [7]. However, the majority of identified SNPs have unknown biological functions and some of these studies yielded contradictory results, suggesting a need for further examination into the functions of identified SNPs related to obesity.

Fat mass and obesity-associated (FTO) variant rs9939609 was the first [8] locus to be positively associated with obesity-related phenotypes [8–12]. FTO is highly expressed in the hypothalamus and liver, appears to function in the central nervous system, and may have a role in energy balance, food intake regulation, and adipogenesis [13, 14]. There may be cross-talk between the FTO protein and leptin, an adipose-derived cytokine implicated in food intake regulation, energy and glucose homeostasis, lipid metabolism, and reproductive function [15]. The influence of leptin on body weight control is mediated by binding to the long isoform of its receptor (LEPR-b), which stimulates gene transcription by activating cytosolic signal transducer and transcription (STAT) proteins [15]. Recent evidence suggests that the LEPR-b-STAT3 signaling pathway may be involved in FTO regulation by restricting energy in the hypothalamus [16] and there is evidence that the leptin receptor (LEPR) variant rs1137101 is positively associated with obesity [17–19].

Leptin acts with hypothalamic receptors to induce satiety by inhibiting the orexigenic neuropeptide Y (NPY)/agouti-related peptide (AgRP) neuronal activity and stimulating the anorexigenic proopiomelanocortin (POMC)/amphetamine-related transcript (CART) neurons [20]. POMC is cleaved into melancortins and is processed to form the α-melanocyte hormone, which exerts catabolic activity via melanocortin-4 receptors (MC4R) to generate a feeling of fullness to suppress appetite [20]. MC4R is a G-protein-linked receptor widely expressed in the hypothalamus and central nervous system, implicated in energy homeostasis and glucose and lipid metabolism [21]. The MC4R variant rs17782313 was the second gene that was positively associated with common obesity traits [22–25]. By contrast, the MC4R variant rs2229616 is negatively associated with obesity [26, 27].

Peroxisome proliferator−activated receptor-gamma (*PPARG*) is another gene that has an important role in obesity. PPARG is a member of the nuclear hormone superfamily, which is involved in adipocyte differentiation and glucose metabolism [28]. There are evidences that PPARG deficiency results in increased leptin levels [28]. The PPARG rs1801282 variant is positively associated with obesity and has been extensively examined in epidemiological studies [29].

The aim of the present study was to assess the independent contributions of LEPR (rs1137101), FTO (rs9939609), MC4R (rs2229616 and rs17782313), and PPARG-2 (rs1801282) polymorphisms for clinically overweight or obesity phenotypes and endocrine-metabolic traits in prepubertal children.

## Methods

### Study design and participants

This descriptive cross-sectional study was part of a larger project (Nutritional, Biochemical, and Genetic Study of an Overweight and Obese Child Population in the Southern Region) approved by the Directorate General of Health, the Ministry of Science and Education of Portugal and by the Ethics Committee of the Hospital Garcia de Orta, according to the principles of Helsinki Declaration. The project was conductd from January 2009 to June 2013 in a population of prepubertal children (based on Tanner stage) recruited from 87 public schools in Lisbon and the Tagus Valley metropolitan region. Initially, 5989 subjects were initially recruited based on the assessment of anthropometric measurements, bioelectrical impedance, biochemical and genetic analysis.

**Fig. 1:**
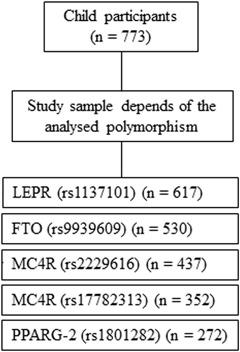
Flowchart of subject participation according to the selected polymorphism

To be included in the study, children should have completed nine years old during the ongoing school year, an inclusion criterion that reduced the initial population to 5577 children. The exact chronological age in days was calculated as the date of examination minus the date of birth. Children who transferred to another school prior to completing the minimum required measurements were excluded, further reducing the population to 5514 eligible children. Children whose parent did not provide written informed consent consent or withdrew consent for venous blood sampling, and those who self-reported as not fasting at the time of blood collection were excluded from the study, further reducing the sample size to 1496 children. For polymorphism analysis, 773 children were enrolled (Fig. 1). To address the possibility of self-selection bias, we compared anthropometric and biochemical data between selected and non-selected participants. No significant differences were found (data not shown).

### Anthropometric and bioelectrical impedance analysis

Clinical assessments were performed in the schools under the supervision of two pediatric consultants. All anthropometric measurements (weight, height, BMI, BMI *z*-score (zBMI), waist circumference (WC), hip circumference (HC), waist-hip ratio (WHR), waist-circumference-to-height ratio (WHtR), mid-upper arm circumference (MUAC), calf circumference (CC), percent body fat (%BF), percent skeletal muscle (%SM), and resting metabolic rate (RMR)) were obtained from barefoot participants dressed with lightweight clothing using methods described previously [30]. zBMI was determined using the least mean squares method [30]. Children were categorized as normal weight (control group) or overweight/obese (case group) per the World Obesity/Policy and Prevention standards [formerly International Obesity Task Force (IOTF)] [31].

### Biochemical analysis

Participants were instructed to fast for 12 hours before venipuncture for blood sampling in the morning at school. Blood samples were refrigerated at approximately 5 °C until transferred to our center immediately processed for serum separation, frozen at −80 °C on the same day, and stored until further analysis. The following serum biochemical parameters were assessed: total cholesterol (TC), high-density lipoprotein (HDL-c), low-density lipoprotein (LDL-c), triglycerides (TG), apolipoproteins A1 (Apo 1) and B (Apo B), glucose, creatinine, total proteins, ferritin, serum insulin and leptin. The homeostasis model assessment of insulin resistance (Homa-IR) was calculated from glucose (mg/dl) and insulin (µU/ml) using the following formula: Homa-IR = (insulin (µU/ml) × glucose (mg/dl))/405. All assessments were made using methods previously described [Furtado JM, Almeida SM, Mascarenhas, P, et al. Anthropometric features as predictors of atherogenic dyslipidemia and cardiovascular risk in a large population of school-aged children. (Under review)].

### Genotyping

Genomic DNA was extracted from a whole peripheral blood sample using the MagNA Pure Compact Nucleic Acid Isolation Kit (Roche Diagnosis, 03730964001) and the MagNA Pure Compact Instrument (Roche Diagnostics GmbH, Germany) per the manufacturer’s instructions. DNA samples were stored at −20 °C until use. Real-time polymerase chain reaction (PCR)was performed in 96-well plates on an automated LightCycler 480 Real-Time PCR System (Roche Diagnostics, Vienna, Austria) using LightCycler FastStart DNA Master Hybprobe (Roche Diagnostics, Berlin, Germany) and LightSNPs (rs1137101 LEPR, rs9939609 FTO, rs2229616 and rs17782313 MC4R, and rs1801282 PPARG-2; TIB Molbiol Synthese labor, Berlin, Germany). The initial step for the allelic discrimination genotyping assay protocol included preincubation at 95 °C for 4 min, followed by 45 cycles of denaturation at 95 °C for 15 s, and annealing, extension, and detection for 40 s at 60 °C. To assess genotyping reproducibility, 10% of the sample was double-genotyped for all SNPs. Concordance rates >99% were obtained for the five tested SNPs. For negative control, Sterile PCR-grade H_2_O was used for the negative control.

### Statistical analysis

SPSS Statistics (IBM, version 24, Armonk, NY, USA) was used for all statistical test procedures. Unless otherwise indicated, variables in tables are means ± standard deviations. Multiple comparisons were made by pairwise *t*-tests with Dunn-Bonferroni adjustment. Pearson’s chi-square statistic was utilized to examine allele/genotype distribution differences across categories and to test for Hardy-Weinberg equilibrium. Phenotype mean differences for each SNP genotype were obtained against the homozygous wild type genotype. Genotype and allele effects on BMI (kg/m^2^) and zBMI for each SNP were calculated according to their formulae as described in Falconer [32]. Major allele dominance type was evaluated using the relationship between dominance and additive effects. Effect size for polymorphic allele on zBMI was graded according to the following cutoffs: very weak effect (|*x*|<0.05), weak effect (0.05<|*x*|<0.2), medium effect (0.8>|*x*|>0.2), and strong effect (|*x*|>0.8). A two-sided *P* < 0.05 determined the significance level for statistical analysis.

## Results

A final sample of 773 Portuguese school children (381 boys and 392 girls) with a mean age of 9.81 years were characterized according to clinical and biochemical parameters. Significant gender-related differences were observed; relative to the values in girls, boys were significantly taller and presented higher mean values of %SM, RMR, HDL-c, Apo A1, glucose, and creatinine, and lower mean values of %BF, LDL-c, TG, Apo B, TC/HDL, LDL/HDL, Apo B/Apo A1, TP, and leptin (*P* < 0.05) (Tables 1 and 2).

**Table 1:** Descriptive clinical characteristics of the study population

	Overall	Gender		IOTF category^a^	
		Male	Female	Normal weight	Overweight/obese
Characteristic	Mean ± SD	Mean ± SD	Mean ± SD	Mean ± SD	Mean ± SD
Age^b, e^	9.81 ± 0.59	9.81 ± 0.61_a_	9.80 ± 0.57_a_	9.81 ± 0.60^c^	9.76 ± 0.59^c^
*Anthropometry*					
Weight (kg)^e^	35.60 ± 8.77	35.91 ± 8.50_a_	35.29 ± 9.02_a_	**31.37** ± **4.95**^c^	**45.39** ± **7.80**^d^
Height (cm)^e^	138.2 ± 7.2	**138.7** ± **6.8**_a_	**137.7** ± **7.4**_b_	**136.9** ± **7.1**^c^	**141.3** ± **6.2**^d^
BMI (kg/m^2^)^e^	18.46 ± 3.37	18.50 ± 3.30_a_	18.42 ± 3.44_a_	**16.66** ± **1.60**^c^	**22.61** ± **2.66**^d^
zBMI^e^	0.65 ± 1.11	0.69 ± 1.07_a_	0.60 ± 1.14_a_	**0.08** ± **0.76**^c^	**1.97** ± **0.47**^d^
WC (cm)^f^	65.7 ± 9.68	65.4 ± 9.3_a_	66.0 ± 10.0_a_	**61.2** ± **5.5**^c^	**76.6** ± **9.0**^d^
HC (cm)^g^	71.0 ± 7.9	71.0 ± 7.8_a_	71.0 ± 8.0_a_	**67.4** ± **5.2**^c^	**80.0** ± **5.9**^d^
WHR (WC/HC)	0.89 ± 0.05	0.90 ± 0.05_a_	0.89 ± 0.05_a_	**0.89** ± **0.05**^c^	**0.92** ± **0.06**^d^
WHtR (WC/height)	0.48 ± 0.06	0.47 ± 0.06_a_	0.48 ± 0.06_a_	**0.45** ± **0.35**^c^	**0.54** ± **0.06**^d^
MUAC (cm)^f^	21.3 ± 3.1	21.3 ± 3.2_a_	21.3 ± 3.0_a_	**19.9** ± **1.9**^c^	**24.8** ± **2.5**^d^
CC (cm)^f^	29.3 ± 3.6	29.4 ±3 .5_a_	29.2 ± 3.7_a_	**27.8** ± **2.6**^c^	**32.9** ± **3.1**^d^
*Bioelectrical impedance*					
BF (%)^f^	21.89 ± 7.96	**21.15** ± **7.36**_a_	**22.63** ± **8.46**_b_	**17.97** ± **5.12**^c^	**31.16** ± **5.17**^d^
SM (%)^f^	31.87 ± 2.85	**32.51** ± **2.78**_a_	**31.25** ± **2.79**_b_	**32.18** ± **2.99**^c^	**31.15** ± **2.39**^d^
RMR (Kcal/day)^f^	1209 ± 114	**1235** ± **120**_a_	**1184** ± **103**_b_	**1169** ± **90**^c^	**1303** ± **13**^d^

Bold values highlights the statistically significant differences between groups

^a^According to World Obesity/Policy and Prevention cut-offs

^b^Age in days presented here as age in years. Distributions (mean ranks) vary between groups with different letters: subscript a, subscript b (*P* < 0.05)

^c^Distributions (mean ranks) and medians are the same between groups (*P* > 0.05)

^d^Distributions are different between groups (*P* < 0.05). Test of significance adjustment was performed using the Dun–Bonferroni correction

^e^*n* = 773, 381, 392, 540, and 233 for overall, M, F, normal weight and overweight/obese

^f^*n* = 661, 329, 332, 468, and 197 for overall, M, F, normal weight and overweight/obese

^g^*n* = 392, 195, 199, 278, and 116 for overall, M, F, normal weight and overweight/obese

*BF* body fat, *BMI* body mass index, *CC* calf circumference, *F* female, *HC* hip circumference, *M* male, *MUAC* mid-upper arm circumference, *RMR* resting metabolic rate, *SM* skeletal muscle, *WC* waist circumference, *WHR* waist-hip ratio, *WHtR* waist- circumference-to-height ratio, *zBMI* BMI *z*-score

**Table 2:** Descriptive biochemical characteristics of the study population

	Overall	Gender		IOTF category^a^	
	(*n* = 625)	Male (*n* = 306)	Female (*n* = 319)	Normal weight (*n* = 425)	Overweight/Obese (*n* = 200)
Characteristic	Mean ± SD	Mean ± SD	Mean ± SD	Mean ± SD	Mean ± SD
TC (mg/dl)	170.1 ± 31.0	169.0 ± 30.2_a_	171.2 ± 31.8_a_	169.8 ± 30.3^c^	171.7 ± 33.2^c^
LDL-c (mg/dl)	90.1 ± 24.4	**87.3** ± **23.1**_a_	**92.7** ± **25.3**_b_	**88.0** ± **23.5**^c^	**95.4** ± **25.9**^d^
HDL-c (mg/dl)	55.5 ± 11.2	**56.5** ± **11.3**_a_	**54.6** ± **10.9**_b_	**57.3** ± **11.5**^c^	**51.4** ± **9.5**^d^
TG (mg/dl)	61.2 ± 27.1	**58.2** ± **27.0**_a_	**64.2** ± **26.9**_b_	**55.7** ± **21.1**^c^	**73.6** ± **34.2**^d^
Apo A1 (g/L)	1.35 ± 0.18	**1.37** ± **0.18**_a_	**1.33** ± **0.17**_b_	**1.37** ± **0.18**^c^	**1.30** ± **0.16**^d^
Apo B (g/L)	0.73 ± 0.18	**0.70** ± **0.17**_a_	**0.76** ± **0.18**_b_	**0.71** ± **0.17**^c^	**0.77** ± **0.19**^d^
TC/HDL	3.14 ± 0.67	**3.07** ± **0.65**_a_	**3.21** ± **0.68**_b_	**3.03** ± **0.61**^c^	**3.40** ± **0.73**^d^
LDL/HDL	1.68 ± 0.55	**1.61** ± **0.53**_a_	**1.75** ± **0.56**_b_	**1.59** ± **0.52**^c^	**1.90** ± **0.56**^d^
Apo B/Apo A1	0.5 ± 0.1	**0.5** ± **0.1**_a_	**0.6** ± **0.2**_b_	**0.5** ± **0.1**^c^	**0.6** ± **0.2**^d^
LDL/Apo B	1.23 ± 0.16	1.24 ± 0.17_a_	1.23 ± 0.15_a_	**1.23** ± **0.17**^c^	**1.24** ± **0.14**^c^
Glucose (mg/dl)	78.5 ± 10.7	**80.2** ± **11.2**_a_	**76.8** ± **9.9**_b_	**77.7** ± **10.5**^c^	**79.8** ± **11.1**^d^
Creatinine (mg/dl)	0.60 ± 0.10	**0.61** ± **0.09**_a_	**0.59** ± **0.11**_b_	0.59 ± 0.10^c^	0.60 ± 0.11^c^
TP (mg/dl)	7.43 ± 0.75	**7.33** ± **0.72**_a_	**7.52** ± **0.76**_b_	**7.36** ± **0.70**^c^	**7.57** ± **0.84**^d^
Ferritin (ng/ml)	37.95 ± 21.32	36.72 ± 16.83_a_	39.12 ± 24.86_a_	37.29 ± 21.47^c^	40.29 ± 21.29^c^
Leptin (ng/ml)^e^	10.08 ± 11.12	**7.71** ± **9.17**_a_	**12.30** ± **12.30**_b_	**5.27** ± **6.32**^c^	**18.99** ± **12.57**^d^
Insulin (µU/ml)^e^	6.88 ± 9.58	6.74 ± 12.58_a_	7.01 ± 5.48_a_	**5.29** ± **4.36**^c^	**9.84** ± **14.69**^d^
Homa-IR	1.29 ± 1.20	1.17 ± 1.10_a_	1.40 ± 1.29_a_	**1.04** ± **0.88**^c^	**1.78** ± **1.56**^d^

Bold values highlights the statistically significant differences between groups

^a^According to World Obesity/Policy and Prevention

^b^Age in days was converted into age in years to compare groups. Distributions (mean ranks) vary between groups with different letters: subscript a, subscript b (*P* < 0.05)

^c^Distributions (mean ranks) and medians are the same between groups (*P* > 0.05)

^d^Distributions are different between groups (*P* < 0.05). Test of significance adjustment was performed using the Dunn-Bonferroni correction

^e^*n* = 330, 160, 170, 211, 119 for overall, male, female, normal weight, overweight/obese, respectively

*Apo A1* apolipoprotein A1, *Apo B* apolipoprotein B, *HDL-c* high-density lipoprotein cholesterol, *LDL-c* low-density lipoprotein cholesterol, *TC* total cholesterol, *TG* triglycerides, *TP* total proteins

Study subjects were stratified by zBMI according to the IOTF category as normal weight (69.9%, *n* = 540) or overweight/obese (30.1%, *n* = 233). Several clinical and biochemical features became progressively poorer as BMI increased, with overweight/obese children presenting significantly higher anthropometric, bioimpedance (except for %SM), and biochemical parameters (except for TC, LDL/Apo B, creatinine, and ferritin) (*P* < 0.05) (Tables 1 and 2).

Four genes were genotyped, with three genotypes identified for each gene, namely: LEPR (AA, AG, GG); FTO (TT, AT, AA); MC4R [GG, GA, AA (rs2229616), TT, TC, CC (rs17782313)]; and PPARG-2 (CC, CG, GG). The three genotypes correspond to homozygous wild type, heterozygous, and homozygous polymorphic, respectively.

The frequencies of wild type (major) and polymorphic (minor) alleles and the genotypes for overweight/obese and normal weight children are presented in Table 3. No statistical differences were found in allele/genotype frequencies between overweight/obese (case) and normal weight (control) subjects for all polymorphisms (*P* > 0.05). The absence of a positive association was extensive, even when the polymorphism effect was analyzed by gender (Table S1) (*P* > 0.05). Genotype frequencies in both overweight/obese and normal weight were in accordance with Hardy–Weinberg equilibrium, except for the LEPR polymorphism in the control group (*P* = 0.0058) (Table 3).

**Table 3:** Allele and genotype frequencies of genetic variant polymorphisms in all subjects, overweight/obese subjects, and normal weight control subjects

Gene	SNP	Allele		*P*	Genotype			Hardy–Weinberg equilibrium test
LEPR	rs11371101	A	G^*^		AA	AG	GG	
	Overall	653 (0.53)	581 (0.47)		190 (0.31)	273 (0.44)	154 (0.25)	**0.006** ^**^
	Overweight/obese	190 (0.56)	152 (0.44)	0.189	52 (0.3)	86 (0.5)	33 (0.19)	0.88
	Normal weight	395 (0.51)	375 (0.49)		115 (0.3)	165 (0.43)	105 (0.27)	**0.0058** ^**^
FTO	rs9939609	T	A^*^		TT	AT	AA	
	Overall	557 (0.44)	483 (0.46)		159 (0.3)	259 (0.49)	112 (0.21)	0.73
	Overweight/obese	183 (0.55)	151 (0.45)	0.752	86 (0.3)	137 (0.47)	68 (0.23)	0.88
	Normal weight	346 (0.54)	298 (0.46)		93 (0.29)	160 (0.5)	69 (0.21)	1.00
MC4R	rs2229616	G	A^*^		GG	GA	AA	
	Overall	865 (0.99)	9 (0.01)		428 (0.98)	9 (0.02)	0 (0.00)	1.00
	Overweight/obese	242 (0.99)	2 (0.01)	0.600	120 (0.98)	2 (0.02)	0 (0.00)	1.00
	Normal weight	557 (0.99)	7 (0.01)		275 (0.98)	7 (0.02)	0 (0.00)	1.00
MC4R	rs17782313	T	C^*^		TT	TC	CC	
	Overall	554 (0.79)	150 (0.21)		222 (0.63)	110 (0.31)	20 (0.06)	0.2
	Overweight/obese	186 (0.82)	42 (0.18)	0.261	76 (0.67)	34 (0.3)	4 (0.04)	1.00
	Normal weight	330 (0.78)	94 (0.22)		132 (0.62)	66 (0.31)	14 (0.07)	0.16
PPARG-2	rs1801282	C	G^*^		CC	CG	GG	
	Overall	495 (0.91)	44 (0.09)		225 (0.83)	45 (0.17)	2 (0.01)	1.00
	Overweight/obese	131 (0.9)	15 (0.1)	0.539	58 (0.79)	15 (0.21)	0 (0.00)	1.00
	Normal weight	342 (0.91)	32 (0.09)		157 (0.84)	28 (0.15)	2 (0.01)	0.63

Bold values highlights the statistically significant differences between groups

*CI* confidence interval

^*^Polymorphic allele

^**^P < 0.05

The mean contribution of different genotypes of each polymorphism (mean (mean 95% CI)) were analyzed for the observed BMI, zBMI, %BF, and biochemical parameters. LEPR rs1137110 was associated with significantly lower BMI (−0.89 (−1.68, −0.09)) and zBMI (−0.24 (−0.50, 0.01)) in the GG genotype, and higher zBMI (0.13 (−0.09, 0.35)) in the heterozygous genotype (Table S2A). The FTO rs9939609 AG genotype was associated with significantly lower TC (−9.35 (−16.44,−2.25)), and the PPARG-2 rs1801282 GG genotype was associated with lower zBMI (−0.24 (−0.50, 0.01)) (Tables S2A and S2B). No other traits were associated with any statistical differences (*P* > 0.05) (Tables S2A and S2B).

To indirectly account for the childhood obesity propensity of each SNP, association analyses between the selected SNPs and zBMI/BMI were conducted (Table 4). Additive effects (half of the divergence between major and minor allele, homozygous outcome) of each selected SNP on zBMI resulted in the homozygous alleles for these selected SNPs were, on average +0.12 (FTO rs9939609), +0.34 (MC4R rs17782313), −0.30 (LEPR rs1137101) and −2.24 (PPARG rs1801282) zBMI units different than major allele homozygous. Conversely, the dominant effects of all SNPs for zBMI are in the opposite direction of the additive ones, reflecting a recessive zBMI inheritance pattern for these SNPs. LEPR rs11371101 and PPARG-2 rs1801282, on average, reduced zBMI / BMI (kg/m^2^) by −0.09/−0.25 and −0.15/−0.21, respectively, with an average decreasing effect of change to minor allele (AECME) of −0.48 and −2.34 kg/m^2^ on BMI, and −0.17 and −1.68 on zBMI, respectively. By contrast, FTO rs9939609 allele contributed, on average, an increase in our sample BMI by +0.06 kg/m^2^ and zBMI by +0.03, with an increase in AECME of +0.12 kg/m^2^ and +0.06 for BMI and zBMI, respectively. The MC4R rs17782313 allele, on average, reduced BMI by −0.12 kg/m^2^, with a decrease in AECME of −0.57 kg/m^2^ on BMI, and accounted for an increase in effect of +0.06 zBMI units with an associated increase in zBMI AECME of +0.26. In general, LEPR and PPARG-2 polymorphisms had weak and medium negative (decreasing) effects on zBMI, respectively, whereas FTO rs9939609 and MC4R rs17782313 had very weak and weak positive (increasing) effects, respectively. However, the effect of PPARG-2 polymorphism was determined by screening only two polymorphic homozygous for zBMI / BMI (kg/m^2^), due to the low relative frequency of the PPARG-2 polymorphic allele in the study population (9%). LEPR and PPARG-2 major alleles (wild type) showed overdominance and partial dominance for zBMI,while the major alleles showed complete dominance for zBMI outcome in FTO rs9939609 and MC4R rs17782313 (Table 4).

**Table 4:** Effects of polymorphisms on BMI and zBMI

	Polymorphism			
	LEPR rs11371101	FTO rs9939609	MC4R rs17782313	PPARG-2 rs1801282
BMI (kg/m^2^)				
Dominant effect	0.52	−0.01	0.05	1.45
Additive effect	−0.43	0.12	−0.54	−1.15
Population mean	18.56	18.7	18.37	18.3
Minor allele average effect	−0.25	0.06	−0.12	−0.21
Average effect of changing to minor allele	−0.48	0.12	−0.57	−2.34
zBMI				
Dominant effect	0.28	−0.07	−0.16	0.68
Additive effect	−0.15	0.06	0.17	−1.12
Population mean	0.81	1.27	1.63	1.24
Minor allele average effect	−0.09	0.03	0.06	−0.15
Average effect of changing to minor allele	−0.17	0.06	0.26	−1.68
Major allele Dominance type on zBMI	Overdominance	Complete dominance	Complete dominance	Partial dominance
SNP effect size on population zBMI	**Weak decrease**	**Very weak increase**	**Weak increase**	Medium decrease*

Bold values highlights the statistically significant differences between groups

*BMI* body mass index, *zBMI* BMI *z*-score

^*^Based on two minor homozygous alleles

We performed an association analysis of anthropometric traits, %BF, and biochemical parameters (dependent variables) on the three different genotypes (independent variable) of the selected genes using zBMI case-control groups (Tables 5 and 6). Significant differences were detected between anthropometric parameters and %BF in the LEPR polymorphism, where homozygous polymorphic normal weight children had significantly lower %BF than wild type homozygous and heterozygous (*P* < 0.05) (Table 5). Significant associations were observed for the MC4R rs17782313 CC genotypes in the overweight/obese group, with significantly higher mean scores for weight, zBMI, WC, WHR, and WHtR, and significantly lower mean scores for BMI, MUAC, CC, and %BF (*P* < 0.05) (Table 5). Higher mean scores in anthropometric parameters (height, BMI, zBMI, WC, HC, WHR, WHtR, MUAC, and CC) and %BF were also found for the FTO rs9939609 AA genotype in the overweight/obese group, whereas lower mean values of BMI, zBMI WC, WHtR, and %BF were observed for the LEPR rs1137110 GG genotype; however, these differences were not statistically significant (*P* > 0.05).

**Table 5:** Comparisons of anthropometric parameters and %BF among all three genotypes in overweight/obese and normal weight subjects

Gene	SNP	Genotype ^1;2,3^(n;n;n)	Weight^1^ (kg)	Height^1^ (cm)	BMI^1^ (kg/m^2^)	zBMI^1^	WC^2^ (cm)	HC^3^ (cm)	WHR (WC/HC)	WHtR (WC/height)	MUAC^2^ (cm)	CC^2^ (cm)	BF^2^ (%)
LEPR	rs11371101												
	Overweight/obese	AA (76;64;36)	47.2 ± 9.1_a_	141.4 ± 6.2_a_	23.29 ± 2.52_a_	2.11 ± 0.47_a_	78.34 ± 8.94_a_	82.4 ± 4.8_a_	0.93 ± 0.07_a_	0.557 ± 0.058_a_	24.8 ± 2.3_a_	33.3 ± 2.6_a_	32.45 ± 4.58_a_
		AG (127;102;65)	46.2 ± 7.9_a_	142.1 ± 6.5_a_	23.58 ± 2.73_a_	2.14 ± 0.47_a_	79.44 ± 9.16_a_	82.5±7.4_a_	0.93±0.05_a_	0.559 ± 0.055_a_	25.4 ± 2.5_a_	34.0 ± 3.4_a_	32.76 ± 5.80_a_
		GG (50;40;24)	45.8 ± 7.4_a_	143.1 ± 5.3_a_	23.18 ± 2.29_a_	2.07 ± 0.47_a_	70.09 ± 7.24_a_	84.0 ± 7.1_a_	0.94 ± 0.08_a_	0.552 ± 0.047_a_	25.6 ± 2.0_a_	33.7 ± 2.8_a_	31.15 ± 5.09_a_
	Normal weight	AA (107;74;16)	31.9 ± 5.1_a_	137.6 ± 7.2_a_	16.95 ± 1.66_a_	0.15 ± 0.78_a_	63.67 ± 5.76_a_	67.8 ± 7.0_a_	0.92 ± 0.04_a_	0.464 ± 0.033_a_	20.2 ± 1.8_a_	28.3 ± 2.3_a_	18.43 ± 4.58_a_
		AG (138;121;32)	31.8 ± 5.1_a_	138.1 ± 7.7_a_	16.89 ± 1.62_a_	0.13 ± 0.76_a_	63.45 ± 5.87_a_	67.1 ± 6.0_a_	0.90 ± 0.04_a_	0.460 ± 0.037_a_	20.3 ± 1.9_a_	28.4 ± 2.4_a_	18.20 ± 5.26_a_
		GG (99;84;16)	31.3 ± 5.0_a_	138.0 ± 7.7_a_	16.59 ± 1.81_a_	-0.05 ± 0.87_a_	62.02 ± 5.85_a_	66.6 ± 6.1_a_	0.90 ± 0.03_a_	0.451 ± 0.040_a_	20.1 ± 2.2_a_	28.1 ± 2.4_a_	**16.89** ± **5.65**_**b**_
FTO	rs9939609												
	Overweight/obese	TT (86;79;61)	45.1 ± 6.9_a_	141.3 ± 6.7_a_	22.81 ± 2.25_a_	2.05 ± 0.42_a_	76.25 ± 8.10_a_	80.5 ± 5.6_a_	0.92 ± 0.06_a_	0.541 ± 0.052_a_	24.7 ± 2.5_a_	32.9 ± 2.8_a_	30.91 ± 5.31_a_
		AT (137;121;94)	45.0 ± 7.8_a_	141.4 ± 6.0_a_	22.89 ± 2.74_a_	2.04 ± 0.46_a_	76.49 ± 8.94_a_	80.9 ± 6.5_a_	0.92 ± 0.06_a_	0.542 ± 0.057_a_	24.8 ± 2.2_a_	32.9 ± 3.3_a_	31.59 ± 5.44_a_
		AA (68;59;46)	46.3 ± 8.3_a_	142.1 ± 6.4_a_	23.24 ± 2.33_a_	2.12 ± 0.40_a_	77.35 ± 8.52_a_	81.7 ± 5.7_a_	0.93 ± 0.06_a_	0.545 ± 0.051_a_	25.1 ± 2.2_a_	33.1 ± 3.1_a_	31.59 ± 5.63_a_
	Normal weight	TT (69;63;31)	31.5 ± 5.2_a_	138.0 ± 7.6_a_	16.85 ± 1.54_a_	0.16 ± 0.74_a_	62.01 ± 5.11_a_	69.7 ± 3.7_a_	0.87 ± 0.04_a_	0.451 ± 0.032_a_	20.0 ± 1.8_a_	28.0 ± 2.4_a_	18.17 ± 5.32_a_
		AT (119;100;62)	30.2 ± 4.9_a_	137.4 ± 7.1_a_	16.99 ± 1.49_a_	0.24 ± 0.71_a_	61.95 ± 5.67_a_	68.7 ± 5.0_a_	0.88 ± 0.05_a_	0.452 ± 0.035_a_	20.2 ± 1.7_a_	28.1 ± 2.3_a_	18.87 ± 4.87_a_
		AA (42;42;15)	31.8 ± 4.5_a_	136.5 ± 7.8_a_	16.56 ± 1.43_a_	0.02 ± 0.72_a_	61.03 ± 3.82_a_	67.5 ± 3.5_a_	0.88 ± 0.04_a_	0.449 ± 0.033_a_	19.8 ± 1.3_a_	28.2 ± 1.7_a_	18.00 ± 5.89_a_
MC4R	rs2229616												
	Overweight/obese	GG (226;204;166)	46.1 ± 8.2_a_	141.7 ± 6.2_a_	23.40 ± 2.77_a_	2.15 ± 0.46_a_	77.7 ± 9.42_a_	81.7 ± 6.0_a_	0.92 ± 0.06_a_	0.549 ± 0.060_a_	25.1 ± 2.4_a_	33.2 ± 3.3_a_	31.90 ± 592_a_
		GA (5;5;3)	52.1 ± 12.5_a_	144.4 ± 8.1_a_	24.05 ± 0.93_a_	2.29 ± 0.19_a_	80.4 ± 6.89_a_	84.0 ± 1.7_a_	0.94 ± 0.06_a_	0.556 ± 0.018_a_	25.9 ± 2.4_a_	35.0 ± 1.4_a_	32.92 ± 5.54_a_
		AA (0;0;0)											
	Normal weight	GG (199;173;84)	30.9 ± 4.9_a_	137.3 ± 7.2_a_	16.59 ± 1.51_a_	0.04 ± 0.73_a_	61.3 ± 4.76_a_	68.5 ± 4.3_a_	0.88 ± 0.05_a_	0.449 ± 0.031_a_	19.7 ± 1.7_a_	27.8 ± 2.1_a_	17.68 ± 5.24_a_
		GA (4;4;4)	33.5 ± 8.3_a_	137.7 ± 9.6_a_	16.25 ± 1.48_a_	-0.10 ± 0.70_a_	60.3 ± 7.43_a_	67.5 ± 1.3_a_	0.84 ± 0.05_a_	0.438 ± 0.027_a_	19.3 ± 2.5_a_	27.9 ± 2.8_a_	16.80 ± 5.68_a_
		AA (0;0;0)											
MC4R	rs17782313												
	Overweight/obese	TT (140;124;91)	47.3 ± 8.0_a,b_	142.6 ± 6.1_a_	23.92 ± 2.75_a_	2.22 ± 0.47_a_	79.5 ± 9.07_a_	83.3 ± 6.1_a_	0.93 ± 0.07_a_	0.559 ± 0.058_a_	25.6 ± 2.4_a_	34.0 ± 3.2_a_	32.93 ± 5.51_a_
		TC (77;69;57)	45.3 ± 6.8_a_	141.6 ± 5.8_a_	**22.69** ± **2.11**_b_	**2.03** ± **0.40**_b_	**75.2** ± **7.98**_b_	**80.3** ± **5.2**_b_	0.92 ± 0.06_a_	**0.532** ± **0.051**_b_	**24.7** ± **2.0**_b_	**32.4** ± **2.8**_b_	**30.82** ± **4.91**_b_
		CC (15;15;11)	57.4 ± 17.1_b_	141.7 ± 7.1_a_	**23.89** ± **4.21**_a,b_	**2.26** ± **0.50**_a,b_	**80.6** ± **12.21**_a,b_	**81.5** ± **5.6**_a,b_	0.94 ± 0.06_a_	**0.568** ± **0.074**_a,b_	**25.2** ± **3.0**_a,b_	**33.3** ± **3.4**_a,b_	**31.35** ± **8.56**_a,b_
	Normal weight	TT (80;67;32)	31.5 ± 5.1_a_	137.3 ± 7.6_a_	16.74 ± 1.37_a_	0.13 ± 0.66_a_	61.3 ± 4.76_a_	68.1 ± 4.2_a_	0.87 ± 0.05_a_	0.448 ± 0.029_a_	20.0 ± 1.5_a_	28.0 ± 2.2_a_	18.09 ± 5.06_a_
		TC (32;24;8)	31.2 ± 4.9_a_	137.0 ± 6.9_a_	16.85 ± 1.77_a_	0.11 ± 0.78_a_	61.2 ± 5.53_a_	70.4 ± 4.2_a_	0.86 ± 0.06_a_	0.450 ± 0.029_a_	19.7 ± 1.9_a_	27.6 ± 2.3_a_	17.60 ± 5.80_a_
		CC (4;4;1)	29.8 ± 6.1_a_	139.5 ± 9.8_a_	16.55 ± 1.49_a_	0.04 ± 0.76_a_	64.6 ± 6.51_a_	71.0^a^	0.82^a^	0.455 ± 0.012_a_	20.8 ± 1.9_a_	29.7 ± 2.1_a_	20.23 ± 5.32_a_
PPAR-	rs1801282												
2	Overweight/ obese	CC (64;64;64)	43.2 ± 6.3_a_	139.0 ± 6.1_a_	22.04 ± 2.16_a_	1.95 ± 0.41_a_	72.70 ± 7.68_a_	79.3 ± 6.2_a_	0.92 ± 0.06_a_	0.522 ± 0.051_a_	23.9 ± 2.1_a_	31.4 ± 2.6_a_	29.96 ± 5.31_a_
		CG (12;12;12)	43.1 ± 7.3_a_	140.9 ± 7.8_a_	21.42 ± 1.18_a_	1.79 ± 0.28_a_	72.54 ± 4.87_a_	79.5 ± 5.2_a_	0.91 ± 0.04_a_	0.515 ± 0.023_a_	24.0 ± 1.3_a_	31.7 ± 2.1_a_	29.40 ± 3.69_a_
		GG (0;0;0)											
	Normal weight	CC (140;140;140)	30.6 ± 4.9_a_	136.8 ± 7.4_a_	16.82 ± 1.55_a_	0.23 ± 0.72_a_	60.31 ± 4.29_a_	68.8 ± 5.7_a_	0.88 ± 0.05_a_	0.442 ± 0.028_a_	20.0 ± 1.9_a_	27.6 ± 2.4_a_	19.29 ± 5.70_a_
		CG (33;33;33)	30.5 ± 4.3_a_	135.3 ± 6.3_a_	16.46 ± 1.58_a_	0.02 ± 0.80_a_	59.48 ± 4.17_a_	67.4 ± 4.8_a_	0.88 ± 0.04_a_	0.440 ± 0.027_a_	19.4 ± 1.9_a_	27.0 ± 2.1_a_	18.23 ± 5.20_a_
		GG (2;2;2)	27.2 ± 2.7_a_	136.9 ± 14.0_a_	17.87 ± 0.19_a_	0.90 ± 0.00_a_	63.00 ± 4.24_a_	72.5 ± 3.5_a_	0.87 ± 0.02_a_	0.461 ± 0.016_a_	21.5 ± 0.7_a_	28.0 ± 2.8_a_	22.75 ± 2.62_a_

Bold values highlights the statistically significant differences between groups

Distributions (mean ranks) are different between groups with different letters: subscript a, subscript b (*p* < 0.05). Test of significance adjustments was performed using the Dunn–Bonferroni correction

^a^This category was not used for comparisons because there was no other category to compare

*BF* body fat, *BMI* body mass index *CC* calf circumference, *HC* hip circumference, *MUAC* mid-upper arm circumference *RMR* resting metabolic rate, *WC* waist circumference, *WHR* waist–hip-ratio, *WHtR* waist circumference-to-height-ratio, *zBMI* BMI *z*-score

^1,2,3^(n;n;n) with n = number of children for each variable

**Table 6:** Comparisons of biochemical parameters among all three genotypes in overweight/obese and normal weight subjects

Gene	SNP	Genotype ^1;2^(n;n)	TC^1^ (mg/dl)	LDL-C^1^ (mg/dl)	HDL-C^1^ (mg/dl)	TG^1^ (mg/dl)	Apo A1^1^ (g/L)	Apo B^1^ (g/L)	Leptin^2^ (mg/dl)	Glucose^1^ (mg/dl)	Insulin^2^ (µU/ml)	Homa-IR
LEPR	rs11371101											
	Overweight/ obese	AA (73;42)	172.9 ± 31.1_a_	98.3 ± 24.2_a_	50.9 ± 9.1_a_	76.2 ± 36.3_a_	1.31 ± 0.14_a_	0.78 ± 0.17_a_	22.13 ± 13.56_a_	78.9 ± 10.9_a_	8.66 ± 7.34_a_	1.89 ± 1.79_a_
		AG (117;62)	172.7 ± 31.4_a_	96.8 ± 27.3_a_	51.1 ± 8.8_a_	77.6 ± 34.2_a_	1.29±0.16_a_	0.76 ± 0.20_a_	19.86 ± 12.58_a_	79.2 ± 11.3_a_	8.98 ± 8.80_a_	1.83 ± 2.03_a_
		GG (46;23)	170.3 ± 35.7_a_	97.6 ± 27.1_a_	50.5 ± 10.0_a_	77.2 ± 32.6_a_	1.30 ± 0.16_a_	0.78 ± 0.21_a_	18.79 ± 9.88_a_	78.3 ± 12.5_a_	18.41 ± 35.27_a_	3.16 ± 7.42_a_
	Normal weight	AA (102;64)	166.2 ± 26.5_a_	83.6 ± 19.4_a_	56.1 ± 10.6_a_	54.8 ± 20.2_a_	1.37 ± 0.19_a_	0.73 ± 0.16_a_	5.19 ± 4.72_a_	73.6 ± 10.3_a_	5.51 ± 4.46_a_	1.13 ± 1.05_a_
		AG (136;80)	166.7 ± 31.2_a_	85.3 ± 24.2_a_	54.9 ± 10.4_a_	56.0 ± 21.0_a_	1.34 ± 0.16_a_	0.70 ± 0.17_a_	5.89 ± 6.53_a_	**76.5** ± **11.4**_**a.b**_	5.39 ± 4.75_a_	0.97 ± 0.79_a_
		GG (99;64)	173.3 ± 36.9_a_	88.5 ± 25.9_a_	56.7 ± 12.7_a_	58.8 ± 22.3_a_	1.37 ± 0.22_a_	0.73 ± 0.19_a_	4.78 ± 4.42_a_	**78.7** ± **10.5**_**b**_	5.62 ± 3.28_a_	1.11 ± 0.76_a_
FTO	rs9939609											
	Overweight/ obese	TT (71;47)	169.4 ± 33.5_a_	94.4 ± 27.6_a_	51.1 ± 9.5_a_	86.3 ± 42.6_a_	1.29 ± 0.15_a_	0.75 ± 0.19_a_	19.15 ± 10.36_a_	79.5 ± 11.9_a_	11.05 ± 18.57_a_	2.86 ± 5.86_a_
		AT (121;74)	172.9 ± 28.0_a_	101.3 ± 24.6_a_	51.5 ± 9.9_a_	**74.1** ± **32.0**_**a.b**_	1.30 ± 0.17_a_	0.79 ± 0.17_a_	18.09 ± 11.46_a_	80.4 ± 11.6_a_	8.10 ± 7.75_a_	1.64 ± 1.75_a_
		AA (65;37)	172.9 ± 28.0_a_	98.9 ± 29.1_a_	51.5 ± 9.4_a_	**71.5** ± **27.4**_**b**_	1.31 ± 0.16_a_	0.77 ± 0.21_a_	19.14 ± 12.74_a_	79.9 ± 10.1_a_	11.04 ± 23.93_a_	1.53 ± 1.45_a_
	Normal weight	TT (69;33)	172.4 ± 29.0_a_	90.7 ± 26.3_a_	58.6 ± 9.4_a.b_	53.7 ± 21.7_a_	1.40 ± 0.16_a_	0.73 ± 0.18_a_	5.63 ± 6.71_a_	75.0 ± 11.0_a_	5.47 ± 4.73_a_	1.09 ± 0.93_a_
		AT (116;60)	166.5 ± 30.0_a_	86.6 ± 21.4_a_	57.4 ± 10.8_a_	55.0 ± 20.2_a_	1.36 ± 0.18_a_	0.70 ± 0.14_a_	5.17 ± 5.17_a_	76.9 ± 10.4_a_	5.09 ± 3.25_a_	0.96 ± 0.66_a_
		AA (41;23)	172.9 ± 33.3_a_	83.7 ± 24.1_a_	62.3 ± 14.5_b_	51.9 ± 21.0_a_	1.44 ± 0.20_a_	0.70 ± 0.20_a_	2.76 ± 1.56_a_	78.7 ± 10.0_a_	5.02 ± 4.87_a_	1.05 ± 1.23_a_
MC4R	rs2229616											
	Overweight/ obese	GG (190;137)	170.5 ± 30.0_a_	99.5 ± 26.6_a_	50.6 ± 9.1_a_	80.0 ± 35.1_a_	1.29 ± 0.15_a_	0.77 ± 0.18_a_	19.52^1^ ± 12.47	80.0 ± 10.5_a_	9.68 ± 16.93_a_	1.95 ± 3.52^a^
		GA (5;1)	186.2 ± 31.1_a_	112.6 ± 31.4_a_	54.4 ± 12.0_a_	79.4 ± 32.5_a_	1.33 ± 0.23_a_	0.83 ± 0.23_a_	12.60^b^	78.8 ± 3.3_a_	4.00^b^	1.26^b^
		AA (0;0)										
	Normal weight	GG (193;106)	169.7 ± 30.3_a_	86.9 ± 22.7_a_	58.1 ± 11.5_a_	55.2 ± 22.0_a_	1.38 ± 0.19_a_	0.71 ± 0.17_a_	4.67±4.32^a^	76.3 ± 10.3_a_	5.34 ± 4.69^a^	0.98 ± 0.88^a^
		GA (4;1)	159.5 ± 27.7_a_	87.5 ± 24.1_a_	55.3 ± 10.3_a_	51.0 ± 11.3_a_	1.36 ± 0.13_a_	0.70 ± 0.13_a_	9.10^b^	73.5 ± 3.9_a_	3.60^b^	0.69^b^
		AA (0;0)										
MC4R	rs17782313											
	Overweight/ obese	TT (125;76)	170.1 ± 30.0_a_	98.9 ± 27.0_a_	50.0 ± 8.6_a_	81.3 ± 35.8_a_	1.28 ± 0.15_a_	0.77 ± 0.18_a_	21.27 ± 12.69_a_	79.3 ± 11.0_a_	11.72 ± 21.80_a_	2.30 ± 4.50_a_
		TC (67;56)	173.1 ± 27.0_a_	99.5 ± 23.9_a_	51.9 ± 10.2_a_	77.3 ± 35.5_a_	1.32 ± 0.17_a_	0.77 ± 0.16_a_	17.07 ± 10.06_a_	80.8 ± 9.5_a_	7.66 ± 6.71_a_	1.64 ± 1.54_a_
		CC (12;8)	160.7 ± 34.5_a_	91.9 ± 29.1_a_	48.3 ± 8.2_a_	73.2 ± 28.7_a_	1.28 ± 0.16_a_	0.70 ± 0.21_a_	19.47 ± 22.67_a_	81.1 ± 9.1_a_	8.15 ± 10.36_a_	1.72 ± 2.56_a_
	Normal weight	TT (79;42)	170.0 ± 32.8_a_	86.4 ± 24.1_a_	59.7 ± 12.0_a_	53.1 ± 20.3_a_	1.41 ± 0.19_a_	0.70 ± 0.18_a_	4.88 ± 6.20_a_	77.3 ± 10.0_a_	5.88 ± 5.37_a_	1.18 ± 1.19_a_
		TC (31;16)	173.7 ± 31.7_a_	87.9 ± 24.4_a_	56.6 ± 8.5_a_	57.3 ± 17.6_a_	1.39 ± 0.15_a_	0.75 ± 0.19_a_	6.27 ± 6.70_a_	78.0 ± 10.1_a_	5.38 ± 3.35_a_	1.09 ± 0.77_a_
		CC (4;2)	169.9 ± 16.4_a_	99.3 ± 11.3_a_	47.0 ± 5.0_a_	73.3 ± 35.8_a_	1.27 ± 0.16_a_	0.78 ± 0.08_a_	6.13 ± 4.20_a_	73.0 ± 10.0_a_	3.03 ± 0.81_a_	0.53 ± 0.23_a_
PPARG-	rs1801282											
2	Overweight/obese	CC (44;34)	170.5 ± 36.0_a_	102.9 ± 28.7_a_	52.5 ± 10.6_a_	75.1 ± 29.2_a_	1.31 ± 0.19_a_	0.78 ± 0.18_a_	18.23 ± 11.64_a_	85.8 ± 22.4_a_	10.48 ± 12.89_a_	2.70 ± 4.54_a_
		CG (8;6)	170.0 ± 21.8_a_	102.9 ± 16.0_a_	52.1 ± 6.4_a_	61.5 ± 21.5_a_	1.29 ± 0.18_a_	0.77 ± 0.12_a_	16.34 ± 2.67_a_	79.1 ± 9.7_a_	6.41 ± 3.75_a_	1.48 ± 0.61_a_
		GG (0;0)										
	Normal weight	CC (101;35)	170.8 ± 24.4_a_	93.0 ± 21.4_a_	60.5 ± 10.3_a_	58.5 ± 22.3_a_	1.39 ± 0.17_a_	0.71 ± 0.13_a_	7.42 ± 12.13_a_	78.4 ± 10.2_a_	3.70 ± 2.76_a_	0.73 ± 0.48_a_
		CG (22;7)	174.2 ± 26.2_a_	96.7 ± 20.9_a_	58.1 ± 8.0_a_	58.2 ± 16.1_a_	1.35 ± 0.14_a_	0.75 ± 0.12_a_	4.02 ± 2.65_a_	77.0 ± 8.2_a_	3.38 ± 2.08_a_	0.67 ± 0.38_a_
		GG (2;1)	181.5 ± 68.6_a_	96.0 ± 11.3_a_	67.0 ± 35.4_a_	54.0 ± 18.4_a_	1.91^a^	0.75 ± 0.06_a_	9.47^a^	80.5 ± 6.4_a_	6.43^a^	1.21^a^

Bold values highlights the statistically significant differences between groups

Distributions (mean ranks) vary between groups with different letters: subscript a, subscript b (*P* < 0.05). Test of significance adjustments was performed using the Dunn-Bonferroni correction

^a^This category was not used for comparisons because there was no other category to compare

^b^This category was not used for comparisons because the sum of case ponderations was less than two

*Apo A1* apolipoprotein A1, *Apo B* apolipoprotein B, *HDL-c* high-density lipoprotein cholesterol, *LDL-c* low-density lipoprotein cholesterol, *TC* total cholesterol, *TG* triglycerides

^1,2^(n;n) with n = number of children for each variable

Regarding the biochemical parameters, children with LEPR rs1137110 AG and GG genotypes in the normal weight group had significantly higher glucose levels than children with AA genotype (*P* < 0.05). Children with FTO rs9939609 AT and AA genotypes in the overweight/obese group had significantly lower TG levels (*P* < 0.05) (Table 6). For the same polymorphism, normal weight subjects with the AA genotype had significantly higher levels of HDL-c (*P* < 0.05) (Table 6).

## Discussion

Ethnicity and environmental factors (i.e., modifying the gene expression but not it’s structure) may affect specific genetic variants under specific conditions, which may distinctly affect obesity-related phenotypes. Obesity can be associated with different metabolic phenotypes of atherogenic lipid profiles and insulin resistance and several studies have investigated the links between obesity, biochemical traits, and polymorphisms to establish possible mechanisms of action [29, 32–34]. This genetic information could be useful to identify children at risk, plan early interventions, and reduce the life-long burden of obesity-related diseases. However, most studies of obesity-SNP associations have yielded controversial results, and the mechanisms underlying the increased risk of obesity conferred by specific alleles remain unclear.

### LEPR rs11371101

The LEPR rs11371101 variant is one of the most frequent LEPR gene polymorphisms and the most likely to have functional consequences [35]. Previous studies reported conflicting results with either positive [21–23] or no association [36] with obesity traits and metabolic parameters. For example, Pyrzaket et al. analyzed a cohort of 101 obese children (12−18 years old) and found that the LEPR gene variant was not associated with obesity, leptin, insulin resistance, or other metabolic abnormalities [36]. Similarly, Endo et al. verified that the LEPR Gln223Arg (rs11371101) polymorphism was not associated with obesity in 553 Japanese school children aged 9−15 years [37]. A meta-analysis of case-control studies and a systematic review also reported that there was no association between the LEPR gene polymorphism and obesity [35, 38]. By contrast, Shabana and Hasnain reported that the LEPR polymorphism was associated with weight, BMI, plasma glucose levels, TC, TG, HDL-c, and LDL-c, whereas it was not associated with WC, HC, and WHR in 475 Pakistani subjects (10–78 years) [39]. Our results show no statistical association between the LEPR polymorphism, anthropometric, and metabolic parameters in normal and overweight/obese children, potentially due to a recessive weak effect on zBMI despite his high frequency (47%) in the studied population.

### FTO rs9939609

FTO functions have not yet been fully established. In an in vitro study, Wu et al. reported that FTO is a co-activator of the CCAAT enhancer-binding protein (C/EBP) family of transcriptional regulators, required in combination with PPARG for adipocyte differentiation, suggesting a role for FTO in the epigenetic regulation of adipose tissue development and maintenance [40]. Several studies reported a positive association between this polymorphism and BMI or other obesity traits in Caucasian populations [8, 10–12], including a study of a few anthropometric parameters by Albuquerque et al. in a cohort of 730 Portuguese children (6−12 years old) [9]. The frequency of the polymorphic allele described by Albuquerque et al. is within the range of our reported values, although the effect of the minor allele on BMI was higher (0.6 kg/m^2^) in the previous study. Studies in Oceanic [41], African [42], and Asian [43] populations found no association between the FTO variant and BMI. By contrast Wu et al. studied Han Chinese adolescents and reported that this FTO variant was positively associated with BMI and metabolic traits such as fasting glucose, insulin, TG, and TC [44]. Conversely, Li et al. found no association between this FTO variant and BMI, WC, %BF, fasting levels of plasma glucose, hemoglobin A1C, insulin, or β-cell function (estimated by homeostasis model assessment) in an adult Han Chinese population [43]. Mangee et al. also found no associations between this FTO variant and the previous biochemical parameters, HDL-c, oxidized LDL, insulin, Homa-IR, and leptin in Austrian (Styrian) adolescents [45]. Consistent with these results, the results of the current study suggest that this polymorphism has no effect, or eventually a recessive very weak increasing one, on zBMI, despite being present at a high frequency (46%) in the studied population.

### MC4R rs2229616 and rs17782313

The MC4R variant is expressed in the central nervous system, and is part of the melanocortin pathway that controls food intake and energy homeostasis [21]. The most common coding MC4R polymorphism is MC4R rs2229616 (V103I missense variant) and it was the first described as showing no association with BMI, plasma insulin, and glucose levels in white British males [46]. Another study reported similar heterozygous frequencies in lean and obese individuals (4.2 vs. 4.5%, respectively), and found no homozygous frequencies for the polymorphic allele [26]. These results are consistent with our findings and indicate that the MC4R rs2229616 polymorphic allele is rare. By contrast, Geller et al. performed a meta-analysis of 7000 individuals and reported that the MC4R polymorphic allele was negatively associated with obesity [47], consistent with the results of other studies [26, 27]. A possible mechanism underlying the protective effect of the MC4R V103I polymorphism could be an increase in energy expenditure [47]. Consistent with others, our results indicate that the MC4R rs17782313 variant is positively associated with obesity traits in overweight/obese children [22–25]. This gene variant may facilitate obesity by increasing the intake of high-energy or fatty foods or promote overeating in response to emotional eating [48]. We did not identify any differences in biochemical parameters associated with different genotypes of this polymorphism.

García-Solis et al. studied 580 children (8−13 years old) and found that heterozygous subjects for this polymorphism risk allele were significantly associated with obesity but not with TC, HDL-c, or insulin levels [22]. Furthermore, Loos et al. confirmed that BMI in children (7−11 years old) was positively associated with each additional copy of the polymorphic allele, with a BMI increase of 0.10 kg/m^2^ and 0.13 *z*-score units (*P* < 7.3 × 10^-6^), twice of that observed in adults (*P* = 0.001) [23] and in agreement with the results of the present study (0.26 zBMI units increase for changing to minor allele). In our population, this recessive increasing effect polymorphism for zBMI yielded a weak effect size. The results from populations of African-American children remain controversial [49].

### PPARG-2 rs1801282

PPARG isoforms 1 and 2 are transcription factors that activate adipocyte differentiation and mediate the expression of specific fat cell genes [28]. However, PPARG-2 rs1801282 may not be associated with obesity and type 2 diabetes *mellitus* [50]. Although we did not detect significant associations, the results showed a trend toward reduced mean BMI in heterozygous overweight/obese children than in homozygous wild type. Furthermore, our results also suggest the presence of a recessive medium decreasing effect on zBMI, although there was a low frequency (9%) of this minor allele in the studied population, which is a limitation to this finding. This is consistent with a study of 194 premenopausal Caucasian Portuguese females, which also found no significant differences in BMI between the control and case groups for this polymorphism [51]. However, a meta-analysis concluded that PPARG variants contributed to human adiposity variation and predisposition for obesity [29]. These inconsistencies may suggest that PPARG polymorphisms may be labeled differently in different ethnic populations, or that there are dissimilar gene-environment interactions.

## Conclusion

We did not detect statistical associations between LEPR rs11371101, FTO rs9939609, MC4R rs2229616, and PPARG-2 rs1801282 polymorphisms and most obesity-related phenotypes and metabolic parameters. Possible explanations could be low statistical power, low carrier frequency, or moderate sample size for some variants. Age differences and genetic environment background could also explain the effect of genes influence in a trait at different developmental stages or the same genes may have a larger impact on a trait as it develops. A longitudinal study may potentially disclose this point, allowing an exploration of the life course genetic associations with clinical and biochemical parameters. Despite these limitations, our data identifies the BMI and zBMI effects of genetic traits that are likely related to obesity, although with modest impact in younger ages, which is in agreement with other authors [9, 23]. This study demonstrates that it is possible to detect and measure the influence of genetic variants on clinical and metabolic characteristics in childhood, reinforcing the concept that there is an important interaction between genes and environment (even if the role of environmental cues may not have much impact in such younger ages) in the development of excessive weight gain and its related complications.

The current study also collected data of weight and height since child birth (Personal Child Health Record) until 9 years of age, which allowed us to determine the adiposity *rebound* (AR) [52] and permitted us to conclude that over 50% of children (data not shown) had an AR prior to the age of 6 years, suggesting that negative environmental factors (e.g., nutritional) are already present in early ages which may explain the high rate of overweight/obesity in our population. Another strength of this study is the characterization of five gene SNPs that were cross-matched with an extensive panel of anthropometric and biochemical parameters. To our knowledge this is the first study trying to establish an association between clinical, metabolic phenotypes and LEPR rs11371101, MC4R rs2229616 and rs17782313 and PPARG-2 rs1801282 in Portuguese children and the first association between biochemical parameters dependent from obesity and FTO rs9939609 in the same population.

Finally, this study showed that MC4R rs17782313 and FTO rs9939609 were positively associated with zBMI, with weak and very weak effects, respectively, suggesting a very scarce contribution to childhood obesity at this age. LEPR rs1137101 and PPARG-2 rs1801282 had weak and medium negative effects on zBMI, respectively, and may slightly protect against childhood obesity. Considering that, in our prepubertal children, the impact on obesity of the SNPs of the genes included in this study is very modest, so we think that, at this age, a clinical application is not justified. Therefore we recommend further research on this topic, with longitudinal design studies or cross-sectional studies including children at a more advanced stage of development taking in account the impact of environmental factors (specially nutritional and physical activity).

## Electronic supplementary material

Supplementary Table S1

Supplementary Table S2A

Supplementary Table S2B
