# Supplementary material for: Association between LEPR, FTO, MC4R, and PPARG-2 polymorphisms with obesity traits and metabolic phenotypes in school-aged children
Source: Endocrine. 2018 Apr 20;60(3):466–78. doi: 10.1007/s12020-018-1587-3 (PMC5937906; doi:10.1007/s12020-018-1587-3)
Supplement: Supplementary file 1 — Supplementary Table S1 [file 12020_2018_1587_MOESM1_ESM.doc]

Table S1. Genotype frequencies of polymorphisms genetic variants in overweight/obese and normal weight subjects stratified by gender.

|  |  |  | Gender | | | | |  |
| --- | --- | --- | --- | --- | --- | --- | --- | --- |
|  |  |  | Male | |  | Female | |  |
| Gene | SNP | Genotype | Overweight/obese | Normal weight |  | Overweight/obese | Normal weight | *P* |
| LEPR | rs11371101 | AA | 20 | 59 |  | 32 | 56 | 0.46 |
|  |  | AG^*^ | 41 | 82 |  | 45 | 83 |  |
|  |  | GG | 16 | 50 |  | 17 | 54 |  |
|  |  |  |  |  |  |  |  |  |
| FTO | rs9939609 | TT | 23 | 45 |  | 28 | 48 | 0.67 |
|  |  | AT^*^ | 43 | 79 |  | 38 | 81 |  |
|  |  | AA | 17 | 38 |  | 18 | 31 |  |
|  |  |  |  |  |  |  |  |  |
| MC4R | rs2229616 | GG | 60 | 129 |  | 60 | 146 | 0.35 |
|  |  | GA^*^ | 1 | 1 |  | 1 | 6 |  |
|  |  |  |  |  |  |  |  |  |
| MC4R | rs17782313 | TT | 36 | 66 |  | 40 | 66 | 0.44 |
|  |  | TC^*^ | 22 | 37 |  | 12 | 29 |  |
|  |  | CC | 1 | 7 |  | 3 | 7 |  |
|  |  |  |  |  |  |  |  |  |
| PPARG-2 | rs1801282 | CC | 27 | 76 |  | 31 | 81 | 0.36 |
|  |  | CG^*^ | 11 | 14 |  | 4 | 14 |  |
|  |  | GG | 0 | 1 |  | 0 | 1 |  |
| CI, confidence interval. ^*^ Polymorphic allele. Significant association when *P* < 0.05 | | | | | | | | |
