# Supplementary material for: Association between LEPR, FTO, MC4R, and PPARG-2 polymorphisms with obesity traits and metabolic phenotypes in school-aged children
Source: Endocrine. 2018 Apr 20;60(3):466–78. doi: 10.1007/s12020-018-1587-3 (PMC5937906; doi:10.1007/s12020-018-1587-3)
Supplement: Supplementary file 2 — Supplementary Table S2A [file 12020_2018_1587_MOESM2_ESM.docx]

Table S2A. Association between polymorphisms and clinical / biochemical response

|  | LEPR rs11371101 | | | | FTO rs9939609 | | | |
| --- | --- | --- | --- | --- | --- | --- | --- | --- |
|  | Genotype | Mean | SE | Mean difference  (95% CI) | Genotype | Mean | SE | Mean difference  (95% CI) |
| BMI  (kg/m^2^) | AA | 18.75 | 0.29 | 0.00 | AA | 18.59 | 0.3 | 0.00 |
|  | AG | 18.84 | 0.23 | 0.08  (-0.61 - 0.27) | AT | 18.69 | 0.21 | 0.09  (-0.62 - 0.80) |
|  | GG | 17.90 | 0.25 | -0.89^*^  (-1.68 - -0.09) | AA | 18.82 | 0.33 | 0.20  (-0.67 - 1.08) |
| zBMI | AA | 0.96 | 0.09 | 0.00 | AA | 1.21 | 0.09 | 0.00 |
|  | AG | 1.09 | 0.07 | 0.13^*^  (-0.09 - 0.35) | AT | 1.20 | 0.07 | -0.01  (-0.23 - 0.21) |
|  | GG | 0.66 | 0.1 | -0.24^*^  (-0.50 - 0.01) | AA | 1.32 | 0.11 | 0.13  (-0.14 - 0.39) |
| % BF | AA | 22.24 | 0.74 | 0.00 | AA | 22.14 | 0.74 | 0.00 |
|  | AG | 22.01 | 0.58 | -0.17  (-1.96 – 1.62) | AT | 22.65 | 0.56 | 0.55  (-1.22 - 2.32) |
|  | GG | 20.85 | 0.68 | -1.37  (-3.43 - 0.69) | AA | 22.16 | 0.79 | 0.08  (-2.09 - 2.25) |
| TC  (mg/dl) | AA | 167.5 | 2.56 | 0.00 | AA | 175.1 | 2.69 | 0.00 |
|  | AG | 171.1 | 2.17 | 3.81  (-2.92 - 10.54) | AT | 165.7 | 2.12 | -9.35^*^  (-16.44 - -2.25) |
|  | GG | 173.2 | 3.03 | 6.28  (-1.39 - 13.95) | AA | 172.7 | 3.73 | -2.51  (-11.00 - 5.98) |
| LDL-c  (mg/dl) | AA | 86.82 | 2.17 | 0.00 | AA | 95.04 | 2.1 | 0.00 |
|  | AG | 88.7 | 1.66 | 2.28  (-2.89 - 7.45) | AT | 89.89 | 1.63 | -4.98  (-10.67 - 0.72) |
|  | GG | 91.43 | 2.16 | 5.28  (-0.61 – 11.17) | AA | 92.75 | 3.28 | -2.00  (-8.83 - 4.82) |
| HDL-c  (mg/dl) | AA | 54.86 | 0.87 | 0.00 | AA | 56.64 | 1.01 | 0.00 |
|  | AG | 54.76 | 0.75 | -0.07  (-2.39 – 2.24) | AT | 54.83 | 0.8 | -1.81  (-4.37 - 0.75) |
|  | GG | 55.13 | 1.05 | 0.44  (-2.20 – 3.08) | AA | 56.19 | 1.23 | -0.59  (-3.65 – 2.48) |
| TG  (mg/dl) | AA | 62.51 | 2.24 | 0.00 | AA | 62.09 | 2.78 | 0.00 |
|  | AG | 62.16 | 1.92 | -0.06  (-5.86 – 5.74) | AT | 60.59 | 2.03 | -1.42  (-8.01 – 5.16) |
|  | GG | 62.27 | 2.51 | 0.17  (-6.53 – 6.78) | AA | 61.67 | 2.87 | -0.18  (-8.06 – 7.70) |
| Leptin  (mg/dl) | AA | 16.61 | 1.34 | 0.00 | AA | 13.36 | 1.63 | 0.00 |
|  | AG | 10.69 | 1.11 | -0.13  (-3.33 – 3.07) | AT | 11.03 | 1.26 | -1.86  (-5.76 – 2.05) |
|  | GG | 8.62 | 1.2 | -2.10  (-5.59 – 1.39) | AA | 10.84 | 1.96 | -1.45  (-6.33 – 3.44) |
| Homa-IR | AA | 1.58 | 0.17 | 0.00 | AA | 1.35 | 0.15 | 0.00 |
|  | AG | 1.32 | 0.14 | -0.28  (-0.66 – 0.11) | AT | 1.28 | 0.15 | -0.10  (-0.53 – 0.34) |
|  | GG | 1.26 | 0.12 | 0.34  (-0.76 – 0.08) | AA | 1.39 | 0.19 | 0.06  (-0.48 – 0.60) |

SE (standard error), Mean difference (mean difference to major allele homozygote). ^*^*P* < 0.05. BF (body fat). BMI (body mass index). HDL-c (High-density lipoprotein cholesterol), LDL-c (Low-density lipoprotein cholesterol), TC (Total cholesterol), TG (Triglycerides) and zBMI (BMI z-score).
