# Supplementary material for: Association between LEPR, FTO, MC4R, and PPARG-2 polymorphisms with obesity traits and metabolic phenotypes in school-aged children
Source: Endocrine. 2018 Apr 20;60(3):466–78. doi: 10.1007/s12020-018-1587-3 (PMC5937906; doi:10.1007/s12020-018-1587-3)
Supplement: Supplementary file 3 — Supplementary Table S2B [file 12020_2018_1587_MOESM3_ESM.docx]

Table S2B. Association between polymorphisms and clinical / biochemical response

|  | MC4R rs17782313 | | | | MC4R rs2229616 | | | | PPARG-2 rs1801282 | | | |
| --- | --- | --- | --- | --- | --- | --- | --- | --- | --- | --- | --- | --- |
|  | Genotype | Mean | SE | Mean difference  (95% CI) | Genotype | Mean | SE | Mean  difference  (95% CI) | Genotype | Mean | SE | Mean  difference  (95% CI) |
| BMI  (kg/m^2^) | TT | 19.21 | 0.26 | 0.00 | GG | 18.52 | 0.18 | 0.00 | CC | 18.08 | 0.21 | 0.00 |
|  | TC | 18.72 | 0.34 | -0.47  (-1.38 - 0.43) | GA | 18.59 | 1.19 | 0.12  (-2.29 – 2.53) | GC | 18.38 | 0.5 | 0.25  (-0.76 - 1.27) |
|  | CC | 18.13 | 1.32 | -1.15  (-2.97 - 0.66) |  |  |  |  | CC | 15.78 | 0.92 | -2.34  (-0.64 - 1.96) |
| zBMI | TT | 1.46 | 0.08 | 0.00 | GG | 1.16 | 0.06 | 0.00 | CC | 1.27 | 0.07 | 0.00 |
|  | TC | 1.47 | 0.1 | -0.01  (-0.26 - 0.25) | GA | 1.23 | 0.45 | 0.15  (-0.64 – 0.94) | GC | 0.82 | 0.16 | -0.47^*^  (-0.82 - -0.11) |
|  | CC | 1.8 | 0.25 | 0.32  (-0.19 - 0.84) |  |  |  |  | CC | -0.98 | 1.08 | -2.33^*^  (-3.87 - - 0.78) |
| % BF | TT | 23.52 | 0.63 | 0.00 | GG | 22.03 | 0.45 | 0.00 | CC | 21.93 | 0.52 | 0.00 |
|  | TC | 22.75 | 0.89 | -0.66  (-2.84 - 1.51) | GA | 21.93 | 2.58 | -0.45  (-5.99 – 5.10) | GC | 21.23 | 1.27 | -0.61  (-3.14 – 1.93) |
|  | CC | 20.52 | 2.7 | -3.44  (-7.77 - 0.88) |  |  |  |  | CC | 20.2 | 0 | -1.47  (-16.64 - 13.71) |
| TC  (mg/dl) | TT | 171.3 | 2.4 | 0.00 | GG | 169,6 | 1.71 | 0.00 | CC | 172.3 | 2.54 | 0.00 |
|  | TC | 171.6 | 3.19 | -0.39  (-8.43 – 7.64) | GA | 175 | 9.33 | 5.62  (-17.00 -28.24) | GC | 168.3 | 4.8 | -3.63  (-14.99 - 7.73) |
|  | CC | 164 | 5.15 | -7.00  (-23.34 - 9.33) |  |  |  |  | CC | 148.5 | 25.5 | -27.75  (-69.89 - 14.39) |
| LDL-c  (mg/dl) | TT | 94.64 | 2 | 0.00 | GG | 91.65 | 1.37 | 0.00 | CC | 97.44 | 2.12 | 0.00 |
|  | TC | 91.22 | 2.37 | -3.02  (-9.48 – 3.44) | GA | 99.29 | 7.02 | 7.01  (-10.99 -25.00) | GC | 91.48 | 4.07 | -5.54  (-14.94 – 3.85) |
|  | CC | 88.5 | 3.85 | -5.60  (-18.73 - 7.54) |  |  |  |  | CC | 81.5 | 3.5 | -19.16  (-54.02 - 15.71) |
| HDL-c  (mg/dl) | TT | 54.53 | 0.8 | 0.00 | GG | 55.43 | 0.63 | 0.00 | CC | 57.93 | 0.98 | 0.00 |
|  | TC | 56.45 | 1.32 | 1.47  (-1.45 – 4.39) | GA | 59 | 2.8 | 4.08  (-4.19 – 12.35) | GC | 59.61 | 1.6 | 1.58  (-2.73 – 5.89) |
|  | CC | 56.36 | 3.2 | 1.92  (-4.02 – 7.85) |  |  |  |  | CC | 47.5 | 16.5 | -11.53  (-27.52 – 4.46) |
| TG  (mg/dl) | TT | 63.42 | 2.34 | 0.00 | GG | 64.56 | 1.55 | 0.00 | CC | 61.5 | 2.3 | 0.00 |
|  | TC | 60.73 | 2.7 | -2.74  (-10.28 - 4.79) | GA | 60.43 | 8.65 | -1.57  (-22.09-18.95) | GC | 54.95 | 4.05 | -6.50  (-16.83 – 3.83) |
|  | CC | 55.21 | 2.59 | -8.09  (-23.39 - 7.21) |  |  |  |  | CC | 71.5 | 34.5 | 10.39  (-27.92 - 48.70) |
| Leptin  (mg/dl) | TT | 12.61 | 1.5 | 0.00 | GG | 11.72 | 1.03 | 0.00 |  |  |  |  |
|  | TC | 12.21 | 2.1 | 0.88  (-4.28 – 6.04) | GA | 9.1 | 0 | -5.16  (-30.62-20.31) |  |  |  |  |
|  | CC | 20.53 | 8.98 | 6.58  (-4.14 - 17.30) |  |  |  |  |  |  |  |  |
| Homa-IR | TT | 1.51 | 0.17 | 0.00 | GG | 1.29 | 0.1 | 0.00 | CC | 1.12 | 0.16 | 0.00 |
|  | TC | 1.18 | 0.12 | -0.34  (-0.86 – 0.17) | GA | 0.69 | 0 | -0.76  (-3.17 – 1.65) | GC | 0.98 | 0.19 | -0.12  (-0.80 – 0.55) |
|  | CC | 2.3 | 1.22 | 0.66  (-0.56 – 1.89) |  |  |  |  | CC | 0.82 | 0 | -0.33  (-2.61 – 1.96) |

SE (standard error), Mean difference (mean difference to major allele homozygote). ^*^*P* < 0.05. BF (body fat). BMI (body mass index). HDL-c (High-density lipoprotein cholesterol), LDL-c (Low-density lipoprotein cholesterol), TC (Total cholesterol), TG (Triglycerides) and zBMI (BMI z-score).
